# Nanotechnologies for Skin Drug Delivery: Polymeric, Bio-Based, and Hybrid Nanocarriers with Clinical and Translational Perspectives

**DOI:** 10.3390/ph19071057

**Published:** 2026-07-08

**Authors:** Lina Eltaib, Hamoud Alotaibi, Mona Al Hamod, Saleh Alfuraih, Noura Al Hamood, Ahmad Mohammad Balkhair, Abdullah Abdulrahman Aljasser

**Affiliations:** 1 Department of Pharmaceutics, Faculty of Pharmacy, Northern Border University, Rafha 73213, Saudi Arabia; 2 Department of Pharmacology and Toxicology, Faculty of Pharmacy, Northern Border University, Rafha 76313, Saudi Arabia; 3 Department of Pharmaceutics, Faculty of Pharmacy, King Khalid University, Abha 62529, Saudi Arabia; 4 Department of Nano-Medicine Research, Institute for Research and Medical Consultations (IRMC), Imam Abdulrahman Bin Faisal University, P.O. Box 1982, Dammam 31441, Saudi Arabia; 5 Department of Pharmaceutics, College of Pharmacy, Imam Abdulrahman Bin Faisal University, P.O. Box 1982, Dammam 31441, Saudi Arabia

**Keywords:** bio-based polymers, polymeric nanocarriers, hybrid nanosystems, skin drug delivery, transdermal delivery, biodegradable biomaterials, controlled release, microneedles, clinical translation

## Abstract

The skin is the largest organ of the human body and acts as a major protective barrier against external agents. However, the highly organized stratum corneum limits the effective delivery of many therapeutic compounds, especially hydrophilic and high-molecular-weight drugs. Conventional topical formulations often exhibit poor permeability, low bioavailability, and limited targeting efficiency. This review discusses recent advances in nanotechnology-based drug delivery systems, including bio-based, biodegradable, and biocompatible polymeric nanocarriers for dermal and transdermal applications, with particular emphasis on vesicular, polymeric, and hybrid nanosystems. Nanocarriers such as liposomes, ethosomes, transfersomes, polymeric nanoparticles, micelles, nanogels, and lipid–polymer hybrid systems have demonstrated improved drug solubility, stability, controlled release, and skin permeation for localized (dermal) delivery compared with conventional formulations. In addition, biodegradable polymeric materials enhance dermal deposition and prolong drug retention, leading to improved therapeutic efficacy. These nanosystems can facilitate enhanced transdermal drug transport under optimized conditions; however, the extent of systemic delivery varies widely depending on drug physicochemical properties, formulation characteristics, and application conditions. Drug transport may occur through intercellular, transcellular, and follicular pathways, resulting in enhanced bioavailability and site-specific delivery. Claims regarding transdermal (systemic) absorption are restricted to cases supported by in vivo or clinical evidence. Furthermore, combining nanocarriers with microneedles and stimuli-responsive platforms has expanded the potential for controlled and on-demand transdermal delivery. Recent preclinical and clinical studies have reported that nanocarrier-based methotrexate gels reduced PASI-like scores by over 70% in psoriatic models, while oleic acid vesicle formulations achieved more than 95% cure rates in rodent models of tinea corporis. Despite these advances, challenges related to large-scale production, stability, regulatory approval, and clinical translation remain significant. Future developments integrating smart nanocarriers, bio-based polymeric biomaterials, wearable technologies, and AI-assisted design may improve personalized dermatological therapies. These innovations in nanocarrier drug delivery are accelerating the translation of advanced therapies to the clinic, promising safer, more effective and personalized dermatological treatments.

## 1. Introduction

The skin is the largest organ of the human body and a formidable physical, chemical and immunologic barrier. The outer layer of the skin, the stratum corneum, is a major barrier to penetration of most xenobiotics, especially of hydrophilic and high-molecular-weight drugs. The barrier function of the skin makes conventional topical formulations (creams, ointments, gels, lotions) inefficient for many indications and largely prevents passive transdermal delivery of macromolecules [1]. Transdermal drug delivery systems (TDDS) are advantageous as they avoid hepatic first-pass metabolism, sustain plasma levels, improve patient adherence and can target drug to diseased skin with reduced systemic exposure. The increasing prevalence of chronic and recalcitrant skin diseases demands the development of advanced drug delivery systems for the skin. Furthermore, poor bioavailability, limited penetration of large or hydrophilic molecules, formulation stability and regulatory issues are some of the major obstacles for the translation of novel nanocarriers from research to clinical use [2]. Nanocarriers like liposomes, ethosomes, transfersomes, solid lipid nanoparticles, polymeric nanoparticles, micelles and nanogels can improve percutaneous permeation, increase drug loading, stabilize labile actives and enable controlled or targeted release for both local and systemic therapy. Recent studies indicate that these systems consistently improve dermal (localized) and systemic transdermal exposure versus conventional formulations in inflammatory dermatoses, infections, psoriasis, acne, alopecia and skin cancers [3].

This review focuses on evaluating three main categories of advanced nanotechnologies for transdermal drug delivery: lipid-based vesicular systems such as liposomes, ethosomes, transfersomes, oleic acid vesicles and niosomes utilize lipid bilayers to interact with or modify the stratum corneum, thereby enhancing drug permeation and inducing local and systemic effects. The extent of stratum corneum modification depends on factors such as lipid composition and the presence of penetration enhancers [4]. However, polymers, including polymeric nanoparticles, micelles and nanogels, are engineered by polymer composition, size, surface charge and stimuli-responsive features to achieve controlled release and precise targeting within skin layers. Hybrid approaches also incorporate lipid and polymer components, often delivered via microneedle arrays, to exploit the barrier-modifying properties of lipids, whilst maintaining the structural stability and functional versatility of polymers, thereby providing true transdermal transport. Therefore, the present study discusses the mechanistic basis of skin penetration, key formulation variables, representative dermatologic indications and translational hurdles such as formulation stability, large-scale manufacturability, batch reproducibility, safety and regulatory pathways that influence therapeutic efficacy and the feasibility of systemic delivery [5]. Although studies have been published so far dealing with specific classes of nanocarriers or specific dermatologic indications, few have been dedicated to comparing polymeric, bio-based and hybrid nanocarrier systems with respect to their clinical and translational prospects. The present study aims to fill this gap by providing a comprehensive synthesis of recent advances, mechanistic insights and translational challenges, thus providing guidance for future research and clinical translation in skin drug delivery.

This review primarily examines polymeric, bio-based and hybrid nanocarriers, including those based on chitosan, hyaluronic acid, alginate, cellulose derivatives and gelatin, for dermal and transdermal drug delivery. However, emphasis is placed on their clinical and translational relevance, including biodegradability, skin compatibility, formulation challenges and regulatory considerations. General descriptions of vesicular systems and transport mechanisms are provided in this study. Therefore, the literature was conducted from major scientific databases, including PubMed, Scopus, Web of Science, Science Direct and Google Scholar, to ensure comprehensive coverage of peer-reviewed research on nanocarrier-based skin drug delivery systems.

## 2. Skin Structure and Barriers Relevant to Nanocarriers

**Figure 1:**
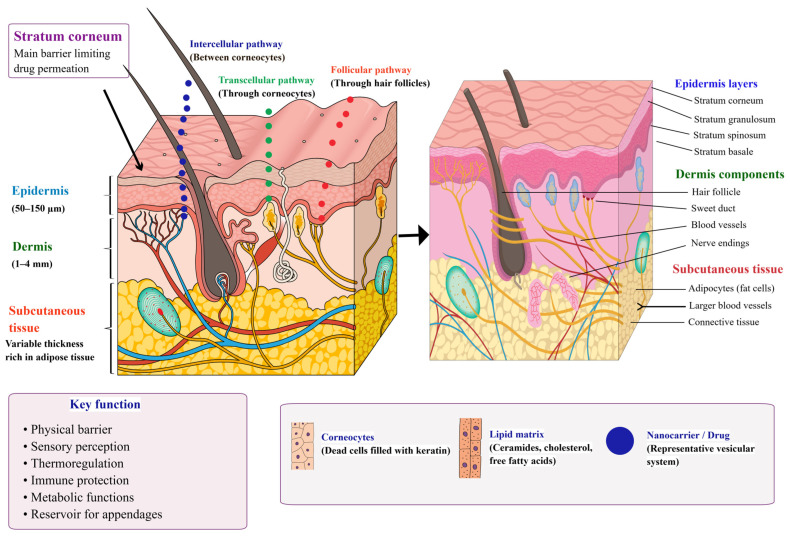
Schematic illustration of human skin highlighting its anatomical structure, functional layers, skin appendages and major penetration pathways relevant to transdermal drug delivery of nanocarrier systems.

The skin has three main layers: epidermis, dermis and subcutaneous tissue. The epidermis is a stratified epithelium composed of the stratum corneum (SC) and viable layers (stratum granulosum, spinosum and basale) and appendages, including hair follicles, sebaceous glands and sweat glands [6]. The dermis contains collagen, elastin and vasculature, provides structural support and is the main site for systemic drug absorption via its capillary network. The subcutaneous tissue consists of adipocytes and larger blood vessels and serves more as a mechanical and thermal buffer than as a diffusion barrier [7]. Moreover, from a drug delivery perspective, the skin is a heterogeneous, multi-layered barrier with the SC as the main rate-limiting layer and the viable epidermis and dermis controlling local and systemic drug effects. Therefore, the design of nanocarriers should be targeted to specific layers of the skin or to bypass barriers to optimize therapeutic outcomes [8]. The skin consists of three major layers, the epidermis, dermis and subcutaneous tissue, as shown in Figure 1. As shown in the figure, the outer stratum corneum acts as the main barrier to penetration of drugs; the viable epidermis and dermis are the main sites for local and systemic absorption of drugs, and the subcutaneous tissue is a supportive layer. Important parts of the skin, such as hair follicles, sweat glands and sebaceous glands, which are important for nanocarrier targeting, are also described, emphasizing the multiple routes for transdermal and dermal drug delivery.

### 2.1. Stratum Corneum as a Barrier and Drug Permeation Pathways

The stratum corneum (SC) is a thin (10–20 μm) yet highly effective diffusion barrier composed of dead corneocytes embedded in a lipid matrix of ceramides, cholesterol and free fatty acids. This tightly organized brick-and-mortar structure confers very low permeability, particularly to hydrophilic and high–molecular weight drugs, thereby limiting passive drug transport across intact skin [9]. Drug permeation across the SC occurs via three principal pathways. The intercellular route involves diffusion through lipid domains between corneocytes and is the dominant pathway for small lipophilic molecules; nanocarriers such as deformable vesicles and lipid nanoparticles enhance this route by interacting with or modifying stratum corneum lipids [10]. The transcellular route requires repeated partitioning across hydrophilic and lipophilic regions of corneocytes, making it less favorable and less commonly exploited by delivery systems [11]. However, the appendageal route consists of transport through hair follicles, sebaceous glands and sweat ducts, bypassing much of the stratum corneum and serving as reservoirs for particulate systems such as liposomes, polymeric nanoparticles and solid lipid nanoparticles, enabling sustained drug release [12].

Conventional transdermal drug delivery systems (TDDS) are largely limited to small (<500 Da), moderately lipophilic molecules, restricting their applicability [13]. In contrast, nanocarriers overcome these limitations through multiple mechanisms. They can fluidize SC lipids to enhance intercellular diffusion, increase hydration and plasticization of the SC via occlusive effects (e.g., SLNs/NLCs), thereby widening diffusion pathways and exploiting follicular targeting for localized and sustained delivery in conditions such as acne and alopecia [14,15,16]. In addition, nanocarriers can act as drug depots within specific skin layers, enabling controlled and prolonged release into the viable epidermis or dermis [17]. Therefore, these mechanisms significantly enhance dermal drug deposition and transdermal flux, leading to improved therapeutic efficacy and reduced systemic toxicity compared with conventional formulations [18].

### 2.2. Impact of Disease and Physiology

The stratum corneum (SC) barrier function is dynamic and influenced by disease, age, anatomical site, hydration and ethnic variability, all of which are critical in the design of nanocarrier-based TDDS. In inflammatory skin diseases such as psoriasis, atopic dermatitis and acne, structural changes (e.g., filaggrin defects, altered lipid organization and increased transepidermal water loss) compromise the barrier, favoring drug and nanocarrier penetration but also increasing the risk of irritation and systemic absorption. Psoriatic lesions are characterized by hyperproliferation and abnormal differentiation of keratinocytes, which may result in local areas of increased permeability [19]. However, SC properties are influenced by physiological factors, e.g., neonatal and elderly skin have higher permeability. Anatomical differences, such as thinner skin on the face and thicker skin on the palms, are important determinants of drug absorption. In addition, hydration and occlusion can increase permeability, which can be exploited by nanocarriers but may also raise safety concerns in compromised skin [20]. Furthermore, variations in SC composition across ethnicities and individuals lead to variations in drug response, which makes it challenging to anticipate nanocarrier behavior and necessitates phenotype-specific optimization [21,22]. Therefore, these changes induced by disease and physiology can facilitate the delivery mediated by nanocarriers, but also decrease the safety margin, highlighting the need for tailored design and careful evaluation of transdermal systems.

## 3. Rationale for Nanocarrier-Based Dermal and Transdermal Drug Delivery

Nanocarrier systems are valuable in dermatological pharmaceutics because they help overcome the poor solubility and limited skin penetration of conventional topical (dermal) formulations. Their tunable features, such as particle size and surface charge, improve stability, stratum corneum interaction and drug delivery performance. They also support controlled, sustained release and can target specific skin layers or cells, making them useful for conditions such as psoriasis and acne, while also enabling non-invasive transdermal delivery that can improve patient compliance and reduce side effects [23]. The mechanistic rationale and key advantages of nanocarriers in enhancing dermal and transdermal drug delivery are summarized in Table 1. Nanocarriers greatly improve dermatological drug delivery through enhanced solubility, penetration, targeting and therapeutic efficacy. They have high drug-loading capacity and protect poorly soluble or unstable drugs such as tacrolimus, methotrexate and vitamin D analogues from degradation and thus improve bioavailability. For instance, solid lipid nanoparticles enhance methotrexate loading and stability, and liposomes have a high encapsulation efficiency for tacrolimus [24].

### Improved Skin Penetration and Deposition

**Figure 2:**
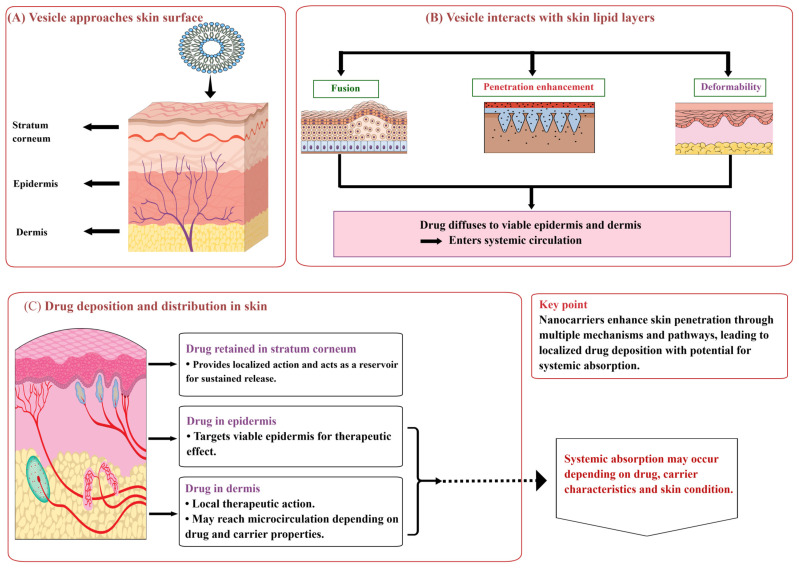
Mechanism of skin penetration of nanocarrier systems. (**A**) Vesicle approaches skin surface; (**B**) initial interaction of nanocarriers with the skin surface and mechanisms facilitating skin penetration by nanocarriers; (**C**) drug deposition and distribution within skin layers with potential for systemic absorption.

Nanocarriers can also enhance skin permeation through interactions with stratum corneum lipids or via follicular pathways. Moreover, it has been shown that ultra-deformable vesicles and hybrid systems increase the permeation and deposition of the drug, resulting in 3–5-fold higher accumulation of the drug in the viable epidermis compared to conventional formulations [24]. They also allow for controlled and sustained drug release, thus decreasing peak plasma levels and prolonging the therapeutic effect; for example, polymeric nanoparticles release drugs over prolonged periods with improved pharmacokinetics [46]. Figure 2 provides a schematic representation of the interaction of nanocarriers with skin layers and mechanisms of their penetration. When applied, vesicles move toward the skin surface and interact with the lipids of the stratum corneum by means of fusion, penetration enhancement and deformability. These processes fluidize or disrupt the lipid matrix, enabling deeper penetration. The drug then permeates the living epidermis and dermis, where it can exert a local therapeutic effect or enter the systemic circulation.

Moreover, nanocarriers can be designed to deliver to specific skin cells or compartments by modulating size, charge and surface ligands, thereby improving therapeutic specificity. Also, functionalized systems such as CD44-targeted nanogels have shown selective drug accumulation in diseased tissues [47]. In addition, these systems localize drug action to the skin, decreasing systemic toxicity and enabling controlled systemic delivery when needed, providing effective therapy at lower doses [48]. Moreover, importantly, nanocarrier-based formulations have frequently demonstrated improved efficacy compared to conventional preclinical models, with some studies reporting enhanced clinical outcomes [49]. Combined, these benefits underline the high potential of nanocarriers to enhance the therapeutic index and clinical performance in dermatologic drug delivery.

## 4. Vesicular Systems

**Figure 3:**
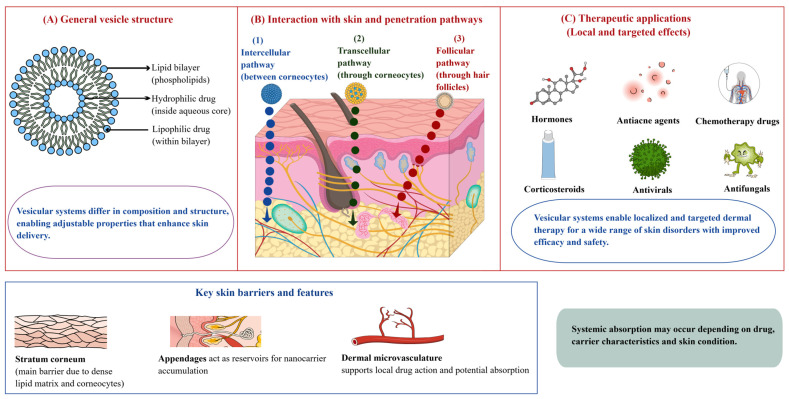
Representative vesicular nanocarrier systems, interaction with skin barriers through multiple penetration pathways and major therapeutic applications for localized and targeted dermal drug delivery.

Nanocarriers for dermal and transdermal drug delivery can be organized into three principal classes based on their composition and structural design: vesicular carriers built from lipid bilayers, polymeric systems with tunable surface chemistry and hybrid or advanced carriers that integrate features from both. Among these, vesicular systems represent the longest-established and most widely investigated platform. Their bilayer architecture enables encapsulation of both hydrophilic and lipophilic pharmacological agents, while membrane flexibility and composition (e.g., ethanol enrichment, surfactant incorporation) allow active modulation of the skin barrier, particularly through fluidization of stratum corneum lipids [50]. Table 2 provides a detailed comparative overview of the three classes, including representative systems, physicochemical features relevant to skin delivery, and their primary therapeutic applications. Figure 3 illustrates the general structural organization of vesicular nanocarriers, showing the lipid bilayer with encapsulated hydrophilic and lipophilic drugs, along with the principal therapeutic domains such as corticosteroids, antivirals, antifungals, hormones, antiacne, and anticancer agents. It further depicts the drug journey from topical application through stratum corneum penetration, epidermal and dermal deposition, and potential entry into systemic circulation.

### 4.1. Conventional Liposomes

Liposomes are spherical vesicles made of phospholipid bilayers and enclosing an aqueous core, allowing simultaneous encapsulation of hydrophilic drugs in the core and lipophilic drugs in the bilayer. Because of their biocompatibility and similarity to biological membranes, they are widely used for cutaneous delivery. However, conventional liposomes are relatively rigid and are mostly restricted to the stratum corneum and upper epidermis, making them more suitable for localized therapy rather than systemic delivery [54]. Liposomal formulations improve drug solubility and stability, especially for poorly water-soluble agents such as vitamin D analogues, corticosteroids and tacrolimus, protecting them from degradation [55]. The physicochemical properties, e.g., size (50–300 nm), surface charge and lipid composition, can be tailored to enhance targeted deposition in the epidermis and dermis, thus improving therapeutic outcomes in inflammatory skin diseases [56]. Furthermore, multilamellar liposomes serve as drug reservoirs, allowing for controlled and sustained release, thereby reducing dosing frequency and limiting systemic exposure [57]. Recent studies have shown that liposomal systems improve skin penetration, retention and anti-inflammatory activity, as demonstrated with estradiol-loaded liposomal gels in models of psoriasis. Moreover, they are widely used for the delivery of anticancer agents and alopecia treatments, offering advantages such as enhanced drug stability and follicular targeting. However, they have critical formulation factors, for example, phospholipid type, cholesterol ratio, vesicle size and surface charge, which play a key role in liposome performance, including skin interaction, elasticity and drug release behavior [58].

### 4.2. Ultra-Deformable Vesicles

#### 4.2.1. Transfersomes

Transfersomes are highly elastic liposomes containing edge activators (like sodium cholate, Tween 80 and Span 80) which destabilize the bilayer and increase deformability. They can thus cross narrow intercellular lipid channels under the action of hydration gradients. This increased permeation is attributed to bilayer fluidization, osmotic driving forces and disruption of SC lipid packing. Transfersomes have been widely used for transdermal delivery of anticancer drugs, NSAIDs, anesthetics and hormones with improved efficacy and reduced systemic exposure compared to conventional formulations [59].

#### 4.2.2. Ethosomes 

Ethosomes are soft phospholipid vesicles with high concentrations of ethanol, which increases the fluidity of the membrane and disrupts the stratum corneum lipid packing pattern to facilitate transdermal delivery. Moreover, they had higher skin penetration than conventional liposomes and hydroethanolic solutions. The fluorescence intensity of Rhodamine B showed that the ethosomes had higher fluorescence accumulation during the study period. Also, ethosomes showed a long residence time in the skin; fluorescence intensities remained higher than in controls even after drug removal. Several studies have demonstrated the superior permeation capability of ethosomal systems compared with conventional liposomes. Touitou et al. demonstrated significantly enhanced skin penetration and transdermal delivery of active compounds using ethosomal carriers, which was attributed to the synergistic effects of ethanol-mediated modification of stratum corneum lipids and vesicle deformability. Enhanced skin deposition and deeper penetration have subsequently been reported for a variety of anti-inflammatory, antifungal and corticosteroid agents [60]. These properties make ethosomes effective carriers for antiviral, anti-inflammatory and hormonal agents with the potential to reduce dosing frequency [61].

#### 4.2.3. Transethosomes

Transethosomes are vesicles with high deformability and increased permeability obtained by combining the ethanol-rich composition of ethosomes with the surfactant-based edge activators of transfersomes. Ethanol disrupts SC lipids, and surfactants increase vesicle elasticity, causing synergistic effects. These systems exhibit higher drug flux and sustained plasma levels than individual vesicular systems and are used for the delivery of analgesics, antihypertensives and dermatological agents [62].

### 4.3. Niosomes and Oleic Acid Vesicles

Niosomes are vesicular carriers formed from non-ionic surfactants (e.g., Span 60, Tween 80) together with cholesterol, offering greater chemical stability, lower cost and enhanced shelf-life relative to phospholipid liposomes. They readily encapsulate hydrophobic drugs such as minoxidil or antifungal agents and can be combined with conventional chemical penetration enhancers to improve cutaneous delivery. Studies have shown that niosomal formulations of minoxidil increase follicular uptake and hair growth efficacy in alopecia models, while niosomal terbinafine demonstrates superior skin flux against dermatophyte infections [63]. However, oleic acid vesicles exploit the fatty acid nature of oleic acid as both a structural component and a penetration enhancer. Oleic acid vesicles disrupt SC lipid organization, thereby increasing the permeation of encapsulated drugs. Vesicles based on oleic acid have been reported to significantly boost the transdermal flux of antifungal agents such as clotrimazole and itraconazole, achieving complete mycological cure in rodent models of tinea corporis. Their dual function as carrier and enhancer makes them especially attractive for topical treatments of dermatomycoses and other infections where high local drug concentrations are required [64]. Natural polymer-based nanocarriers, such as chitosan, hyaluronic acid, alginate, cellulose derivatives and gelatin, have shown promise as platforms for dermal and transdermal drug delivery. They have good biocompatibility, biodegradability and skin compatibility, improving drug penetration, retention and controlled release. In addition, their utilization in wound healing, inflammatory skin diseases and targeted drug delivery exhibits immense translational value; however, more clinical studies are required for their widespread therapeutic use, as highlighted in Table 3.

## 5. Polymeric Nanocarriers

Polymeric nanocarriers offer a versatile platform for dermal and transdermal drug delivery, distinguished by their tunable physicochemical attributes that can be engineered for specific skin targets. These systems overcome many limitations of conventional formulations by enhancing drug solubility, providing protection against degradation and enabling controlled release [71].

### 5.1. Polymeric Nanoparticles

Polymeric nanoparticles (PNPs) are solid colloidal systems, typically ranging from 50 to 500 nm, composed of biodegradable polymers such as poly (lactic-co-glycolic acid) (PLGA), poly (lactic acid) (PLA), polycaprolactone or natural polymers like chitosan and alginate. PNPs can be designed as nanospheres, where the drug is dispersed within the polymer matrix, or as nanocapsules, featuring a drug-containing oily or aqueous core encased in a polymer shell [72]. For dermal therapeutic applications, PNPs offer several key advantages such as high encapsulation capacity for poorly soluble drugs, protection of encapsulated agents from enzymatic degradation, controlled as well as sustained release kinetics and the ability to functionalize surfaces with polyethylene glycol (PEG) or targeting ligands to optimize skin interaction and cellular uptake [73]. These properties have been exploited in several dermatological conditions; for example, in psoriasis, PNPs loaded with methotrexate, calcipotriol, corticosteroids or biologics have shown improved anti-psoriatic efficacy and reduced systemic toxicity in preclinical models. PNPs containing clindamycin, chloramphenicol or spironolactone enhance follicular targeting and reduce local irritation in acne and rosacea. However, in skin cancers, PNPs carrying 5-fluorouracil, imiquimod or photodynamic agents can penetrate the tumor more deeply and achieve a higher local drug concentration. A specific in vivo review found that both polymeric and inorganic nanoparticles improve the efficacy of drugs in animal models of dermatitis due to enhanced skin retention and cellular uptake [74,75].

### 5.2. Polymeric Micelles

Polymeric micelles are self-assembled nanostructures formed from amphiphilic block copolymers, such as PEG-PLA and PEG-PCL, characterized by a hydrophobic core that solubilizes lipophilic drugs and a hydrophilic shell that stabilizes them in aqueous media. These characteristics make them highly effective for increasing the solubility and stability of poorly water-soluble drugs [76]. In cutaneous drug delivery, polymeric micelles offer several benefits. They increase the solubility and stability of lipophilic drugs like calcineurin inhibitors and retinoids. They can enhance drug partitioning into the skin and, depending on their size and surface properties, facilitate deeper penetration [77]. Furthermore, polymeric micelles can be readily incorporated into gels or patches, improving patient acceptability and adherence. A dedicated study highlights the significant potential of polymeric micelles in treating various conditions affecting normal and diseased skin, including atopic dermatitis, psoriasis and skin infections. Polymeric micelles are self-assembled nanostructures formed by amphiphilic block copolymers, e.g., PEG-PLA, PEG-PCL, with a hydrophobic core and hydrophilic shell. They excel at solubilizing highly hydrophobic drugs and stabilizing them in aqueous media. In cutaneous delivery, polymeric micelles increase the solubility and stability of lipophilic drugs (e.g., calcineurin inhibitors, retinoids). Also, they enhance partitioning into skin and sometimes facilitate deeper penetration depending on size and surface properties. When combined with gels or patches, they improve patient acceptability. Studies highlight polymeric micelles for cutaneous drug delivery and outline their promise in normal and diseased skin, including atopic dermatitis, psoriasis and skin infections [78].

### 5.3. Chitosan-Based Nanogels for Dermal Delivery

Nanogels are nanoscale crosslinked hydrogel systems that offer a balance of high drug loading, tunable release and colloidal stability. They can be designed to be pH-, temperature-, or enzyme-responsive, which allows targeted drug release in diseased microenvironments [79]. Among them, thermosensitive nanogels undergo sol–gel transitions in response to temperature changes, so they can remain liquid during storage or application and form gels upon reaching skin temperature, thus providing controlled and localized drug release. For example, polymers like poly(N-isopropylacrylamide) (PNIPAM) are commonly used for such systems. On the other hand, pH-sensitive nanogels are formulated to respond to the acidic or basic environments characteristic of inflamed or diseased skin. These nanogels, often based on chitosan or poly (acrylic acid), may also contract in response to pH shifts, allowing site-specific drug release in conditions such as infections or psoriasis. However, the selection of thermosensitive or pH-sensitive nanogels is tailored for improved drug delivery according to the physiological environment and clinical applications [80,81]. These systems have demonstrated improved therapeutic efficacy and reduced systemic exposure in dermatology, cosmetics and dermal cancer therapy. Overall, polymeric nanocarriers represent a highly tunable platform in which size, surface charge, hydrophilicity and degradability can be optimized for targeted skin delivery.

## 6. Hybrid and Advanced Nanocarrier Systems

### 6.1. Lipid-Based and Lipid–Polymer Hybrid Nanocarriers

Solid lipid nanoparticles (SLNs) and nanostructured lipid carriers (NLCs) are lipid-based colloidal carriers usually in the range of 50–300 nm. In SLNs, drugs are incorporated into a solid lipid matrix, whereas in NLCs, drugs are incorporated into a mixture of solid and liquid lipids. The key distinction lies in the internal structure of SLNs, which possess a highly crystalline, ordered lipid matrix that can limit drug loading capacity and increase the risk of drug expulsion during storage as the lipids crystallize further. On the other hand, NLCs are designed with a mixture of solid and liquid lipids, producing an imperfect or less ordered matrix with more lattice defects. This imperfect structure allows for higher drug loading and improved stability; in addition, it reduces the tendency for drug expulsion, making NLCs especially suitable for the delivery of poorly soluble or unstable drugs [82]. SLNs and NLCs are advantageous for dermatological applications. Their lipidic nature allows them to form an occlusive film on the surface of the skin, reducing transepidermal water loss, increasing the hydration of the stratum corneum and improving skin permeability [83]. Moreover, the lipid matrix can interact and fluidize stratum corneum lipids, thus enhancing dermal deposition and penetration of poorly soluble therapeutic agents into the viable epidermis and upper dermis [84]. The nanocarriers are highly biocompatible as they are composed of safe physiological lipids (glyceryl behenate, cetyl palmitate) and can therefore be incorporated into creams, gels, sprays and transdermal patches with low irritation potential [85].

SLNs and NLCs have been reported to provide better therapeutic efficacy in the treatment of psoriasis, acne, infectious skin diseases and alopecia compared with conventional creams and drug solutions in preclinical and early clinical studies. Consistent reports have described improved skin retention, higher local drug concentrations and better clinical responses [86]. Another advanced platform is lipid–polymer hybrid nanoparticles, which combine the advantages of both polymeric and lipid-based systems. These carriers usually have a polymeric core (e.g., PLGA) that offers structural stability and controlled release of the drug, surrounded by a lipid shell that enhances biocompatibility and interaction with biological membranes. Surface modification with PEG, ligands or stimuli-responsive linkers also allows for targeted and controlled drug delivery. Originally developed for systemic administration, these hybrid systems are now increasingly being adapted for skin therapy in order to provide sustained release and better interaction with stratum corneum and hair follicles [87].

### 6.2. Microneedle-Assisted Nanocarrier Delivery

**Figure 4:**
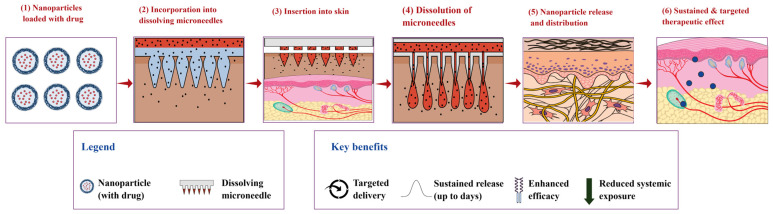
Microneedle-assisted nanocarrier delivery for transdermal drug administration.

Microneedle (MN) technology has been identified as an effective strategy to overcome the barrier function of stratum corneum. MN arrays create temporary microchannels in the skin and greatly enhance the permeation of small molecules and macromolecules with minimal pain and tissue damage. The synergistic effect of coating or dissolving microneedles with nanocarriers is achieved in a way that microneedles allow direct delivery into the viable epidermis or dermis and nanocarriers offer sustained, localized and targeted drug release [88]. A typical example is the dissolving microneedles loaded with hyaluronic acid–methotrexate nanoparticles for the treatment of psoriasis, as illustrated in Figure 4. Hyaluronic acid–methotrexate conjugates self-assemble into nanoparticles that target CD44 receptors overexpressed on inflammatory keratinocytes and immune cells. This nano-in-micro delivery system was able to provide sustained drug release up to seven days, significantly reduce psoriatic lesions in animal models and limit systemic methotrexate exposure. Platforms with similar microneedles and nanoparticles are currently being explored for atopic dermatitis, intradermal vaccination and skin cancer treatment, leading to sustained therapeutic effects, better patient adherence and less frequent dosing [89].

### 6.3. Smart and Stimuli-Responsive Nanocarriers

Smart nanocarriers are sophisticated delivery vehicles that respond specifically to pathological conditions in diseased skin tissues to achieve site-specific, controlled drug release. Reactive oxygen species (ROS)-responsive nanocarriers have emerged as promising platforms for inflammatory skin disorders because elevated oxidative stress within diseased tissues can trigger selective drug release. Likewise, pH-responsive polymeric systems exploit local changes in tissue acidity to achieve site-specific delivery and controlled release, thereby minimizing off-target exposure and improving therapeutic efficacy [90]. Such systems improve the accuracy of the therapy with decreased off-target effects and systemic toxicity. Common strategies are the use of pH- or enzyme-responsive nanocarriers. A pH-responsive nanogel based on chitosan containing curcumin has recently been evaluated in a clinical trial for oral lichen planus, a chronic inflammatory mucocutaneous disease. The nanogel enabled targeted drug release in the acidic environment of inflamed tissue and resulted in significant reductions in disease activity scores compared to standard treatment (ClinicalTrials.gov Identifier: NCT06932848). In the same way, ROS-responsive nano-carriers based on hyaluronic acid–methotrexate have shown promising results in preclinical models of psoriasis, and initial clinical trials are in progress to evaluate their application for the management of chronic inflammatory skin diseases. The extracellular pH of inflamed or cancerous skin tissues is often slightly acidic, and the levels of enzymes such as matrix metalloproteinases are elevated. Thus, nanoparticles bearing acid-labile bonds or enzyme-sensitive peptides can selectively degrade within these abnormal microenvironments and release their payload [91]. Another important class of smart systems is reactive oxygen species (ROS)-responsive nanocarriers. Chronic wounds, psoriasis and some skin cancers are characterized by high levels of ROS. ROS-responsive polymers such as thioketal-based materials are degraded under oxidative stress, allowing rapid and site-specific drug release only at diseased sites [92]. Recent advances are directed toward the combination of stimuli-responsive nanocarriers with wearable biosensors capable of monitoring physiological parameters (temperature, pH and electrical impedance) in real time. Such integrated systems can modulate the drug release according to the disease status, opening the door to closed-loop and personalized transdermal therapy platforms with improved therapeutic efficiency and patient convenience [93].

## 7. Localized Nanocarrier-Based Dermatologic Therapy

Localized dermatologic therapy has been extensively investigated using nanocarrier systems to increase drug accumulation in diseased skin and reduce systemic exposure. Liposomes, solid lipid nanoparticles (SLNs), polymeric nanoparticles, polymeric micelles and hyaluronic acid nanoparticle-loaded microneedles have been used to deliver drugs such as methotrexate, calcipotriol, tacrolimus, siRNA and estradiol in psoriasis, leading to reduced PASI-like scores, inhibition of Th-17 cytokines (IL-17A and IL-23) and improved histopathological results in imiquimod-induced models [94]. Hyaluronic Acid–Methotrexate Microneedle (HA-MTX MN) patches demonstrated good therapeutic efficacy in the IMQ-induced psoriasis mouse model, reducing PASI-like scores compared with untreated and free MTX-treated groups. The total PASI score decreased in the untreated model group after HA-MTX MN treatment, resulting in a reduction in disease severity. In addition, the treatment reduced the expression of pro-inflammatory cytokines, including IL-23, IL-17 and IL-6, indicating effective suppression of psoriatic inflammation [25].

SLNs, nanostructured lipid carriers (NLCs) and polymeric nanoparticles loaded with corticosteroids or calcineurin inhibitors improve epidermal retention, offer controlled drug release, reduce required doses and minimize steroid-induced skin atrophy in dermatitis and eczema [95]. However, transfersomes, ethosomes and polymeric nanoparticles improve the follicular delivery of antibiotics like clindamycin and anti-androgenic agents such as spironolactone in acne and rosacea, increasing local efficacy and reducing systemic absorption and antimicrobial resistance [96]. Liposomal and oleic acid-based vesicular systems have also shown increased penetration of antifungal agents into the stratum corneum and hair follicles, having advantages in resistant dermatophyte infections such as tinea unguium [97]. In the case of skin cancer therapy, liposomes, transfersomes and polymeric nanoparticles loaded with 5-fluorouracil, imiquimod or even photosensitizers are able to reach higher accumulation of the drug in the tumor while showing lower systemic toxicity, showing higher efficacy in basal cell carcinoma, actinic keratosis and melanoma models [98]. A systematic review also showed that organic nanocarriers, especially those based on lipids and polymers, displayed better skin tolerance and more consistent anti-inflammatory effects than inorganic nanoparticles in dermatitis models [99,100]. Representative therapeutics and their advantages over conventional dosage forms are highlighted in Table 4.

### Systemic Therapy Through Transdermal Route

Apart from topical skin therapy, nanocarrier systems have also been investigated for systemic drug delivery through the transdermal route. Ethosomes, transfersomes and transethosomes improve drug partitioning into the stratum corneum and increase the transdermal flux of small-molecule drugs such as antihypertensives and analgesics [106]. Additionally, nanocarriers protect peptides, proteins and nucleic acids from enzymatic degradation and, in combination with physical enhancement methods like microneedles, iontophoresis, electroporation or ultrasound, enhance their transport across the otherwise impermeable skin barrier [107]. Moreover, sustained release nanocarrier systems provide long-acting needle-free therapy for chronic diseases like chronic pain, cardiovascular diseases and neurodegenerative diseases [108]. Recent reviews pointed out that the combination of nanocarriers with physical enhancement methods and wearable sensor technologies significantly enhances macromolecular permeation and provides smart, on-demand systemic drug delivery for precision therapy [109]. Advantages over conventional systemic administration are highlighted in Table 5.

## 8. Critical Formulation Parameters and Characterization

### 8.1. Physicochemical Parameters

Physicochemical attributes of nanocarriers largely determine their interaction with the skin barrier and, ultimately, their therapeutic performance. Particle size and size distribution are particularly important; most dermal nanocarriers fall within the range of approximately 10–500 nm, and smaller, more monodisperse systems (low polydispersity index) generally exhibit more efficient penetration and more predictable biodistribution within the stratum corneum and appendages. Surface charge, usually assessed as zeta potential, also plays a critical role. Cationic systems such as chitosan-based nanoparticles can strongly interact with negatively charged skin components, promoting adhesion and sometimes enhancing permeation, but they may also increase irritation or sensitization risk; consequently, neutral or slightly anionic formulations are often preferred for chronic or large-area applications [114].

The chemical composition and membrane fluidity of the carrier govern its deformability and preferred penetration pathway. In lipid vesicles, the choice of lipid, incorporation of ethanol or edge activators and the degree of acyl chain saturation dictate bilayer fluidity and thus the ability to traverse intercellular lipid domains [115]. In polymeric systems, the balance between hydrophilic and hydrophobic segments, crosslinking density and glass-transition temperature controls matrix rigidity and drug release behavior. High drug-loading capacity coupled with tunable release kinetics is essential for clinical feasibility; modern lipid and polymer matrices are therefore engineered for slow, sustained delivery that can maintain therapeutic levels in the skin over extended periods while minimizing systemic exposure [116]. Dynamic light scattering (DLS) is the most commonly employed technique to determine hydrodynamic diameter, polydispersity index and diffusion coefficient in dispersion. A typical DLS profile might reveal a mean hydrodynamic diameter of approximately sim 23 nm but with multiple intensity-weighted peaks, indicating a multimodal size distribution; such heterogeneity must be minimized through formulation optimization because it can compromise reproducibility, penetration behavior and regulatory acceptability [117].

### 8.2. In Vitro and Ex Vivo Evaluations

Comprehensive in vitro and ex vivo characterization is essential before in vivo testing. In vitro release studies, performed in physiologically relevant media, are used to define release profiles (e.g., burst versus sustained release) and to relate these kinetics to polymer degradation or lipid reorganization [118]. Ex vivo permeation and skin-deposition experiments typically employ human or animal skin mounted on Franz diffusion cells, enabling quantification of drug levels in the stratum corneum, viable epidermis, dermis and receiver compartment; these studies are central for comparing nanocarriers with conventional formulations and for establishing dose–flux relationships [119]. Imaging methods such as confocal laser scanning microscopy, transmission electron microscopy and multiphoton microscopy are widely used to visualize nanocarrier localization, depth of penetration and stratum–corneum ultrastructural changes (e.g., lipid disordering or corneocyte swelling) after treatment [120]. Parallel irritation and cytotoxicity assays, often employing keratinocyte and fibroblast monolayers or reconstructed 3D skin models, assess cell viability, inflammatory markers and barrier integrity following exposure to candidate formulations. Reviews focusing on dermal lipid nanoparticles emphasize systematic monitoring of skin hydration, transepidermal water loss (TEWL), erythema and histological changes after repeated application to ensure that enhanced delivery is not achieved at the expense of barrier damage or long-term irritation [121].

### 8.3. In Vivo Pharmacology and Safety

In vivo evaluation generally relies on rodent models of inflammation, psoriasis, infection or skin cancer, in which nanocarriers are compared with conventional topical or systemic therapies. Endpoints commonly include clinical severity scores, lesion thickness, erythema, pruritus, histopathological grading and quantification of cytokines and other inflammatory mediators [122]. Across multiple models, nanocarrier-based systems tend to enhance therapeutic responses at equal or lower nominal doses, reflecting improved skin retention, cellular uptake and sometimes targeted delivery to specific immune or tumor cell populations [123]. Immunogenicity may involve innate and adaptive immune responses and is commonly evaluated by measuring cytokine concentrations, e.g., interleukin-6 (IL-6), tumor necrosis factor-alpha (TNF-α) and interleukin-1β (IL-1β) after exposure to nanocarriers. In addition, nanocarriers may interact with pattern recognition receptors (e.g., Toll-like receptors, TLRs) on immune cells while activating intracellular pathways like NF-κB, resulting in increased secretion of pro-inflammatory cytokines. For instance, the same cationic lipid nanoparticles have been reported to elevate IL-6 and TNF-α production in keratinocytes and macrophages, underscoring the importance of immunological profiling during nanocarrier development [124]. Systemic drug levels and off-target toxicity are also assessed, and polymeric or lipid nanoparticles frequently show reduced systemic exposure and toxicity compared with oral or injectable routes, particularly for agents such as methotrexate or potent corticosteroids. Most lipid and polymeric nanocarriers are well tolerated on intact skin; however, long-term safety and immunological effects require careful monitoring, especially for cationic formulations, inorganic particles or systems incorporating strong penetration enhancers. High-level reviews of transdermal drug delivery and dermatologic nanomedicine stress the need for standardized, translationally relevant animal models, harmonized reporting of safety and tolerability including (TEWL, immunogenicity and systemic accumulation) and better correlation between preclinical outcomes and human clinical endpoints [125]. In addition to local irritation and cytotoxicity, immunogenicity remains an important consideration for nanocarrier-based systems. Certain nanoparticle formulations may activate macrophages, dendritic cells and complement pathways, resulting in increased production of inflammatory mediators including tumor necrosis factor-alpha (TNF-α), interleukin-1 beta (IL-1β) and interleukin-6 (IL-6). Surface chemistry, particle size, charge and biodegradation products can significantly influence these immune responses. Therefore, comprehensive immunotoxicity evaluation should be incorporated into the preclinical development of advanced dermal nanocarriers [125].

## 9. Translational Challenges and Clinical Prospects

Despite great advances in preclinical studies, only a few nanocarrier-based dermatologic formulations are available in the market as prescription therapies [126]. Major translational challenges are related to large-scale manufacturing and stability, since maintaining particle size, polydispersity and encapsulation efficiency during GMP-compliant production is still technically challenging. Issues such as aggregation, lipid crystallization and polymer degradation can reduce product stability and shelf life, necessitating robust manufacturing methods such as high-pressure homogenization, microfluidics and spray-drying [127].

Regulatory and safety issues are also important aspects, as nanomedicines require extensive physicochemical characterization, evaluation of degradation products and long-term safety studies. Potential risks include accumulation of non-degradable materials, immunogenicity, skin irritation and inadvertent systemic distribution. The absence of standardized classification and risk-assessment frameworks for nanocarriers further complicates regulatory approval [128]. Another challenge is the biological variability among patients, where differences in skin thickness, lipid composition, microbiota and barrier integrity can significantly influence the penetration of nanocarriers and drug release. Disease-specific changes such as hyperkeratosis in psoriasis or impaired barrier function in eczema [129] may alter therapeutic performance and complicate dose optimization. Although a multitude of nanocarrier systems have shown improved efficacy in in vitro and animal studies, robust randomized clinical trials in human subjects remain scarce. At present, most of the commercialized products are cosmetic or over-the-counter formulations, and only a few prescription lipid-based nanocarrier systems are in clinical evaluation for diseases like psoriasis [130]. The successful translation of nanocarrier-based dermatologic therapies into routine clinical practice will, in general, depend on the successful overcoming of manufacturing, regulatory and clinical validation challenges through collaboration among formulation scientists, clinicians and regulatory agencies [131]. Clinical trials in skin drug delivery in dermatology are summarized in Table 6.

## 10. Future Directions

Emerging research is reshaping skin drug delivery by merging nanotechnology with data-driven design, smart devices and biologically inspired materials. AI-guided formulation design and machine learning algorithms are now being applied to large libraries of nanocarrier data (composition, size, surface chemistry, release profiles) to predict optimal formulations for a given active ingredient and target skin layer. By iteratively training on experimental outcomes, AI can reduce the number of trial-and-error batches, accelerate scale-up and identify non-obvious parameter combinations that maximize permeation while preserving stability [132]. Smart, integrated TDDS—The next generation of transdermal systems couples nanocarriers with wearable sensors that continuously monitor physiological cues (e.g., temperature, pH, inflammatory biomarkers). Integrated micro-pumps or electro-responsive polymers can then modulate drug release in real time, creating a closed-loop therapy that delivers drug only when a disease flare is detected [133].

Advanced nanogels and biopolymers, such as chitosan and hyaluronic-acid-based nanogels, have gained attention for their intrinsic muco-adhesive and wound-healing properties. When fabricated as microneedle patches, these nanogels can provide sustained, localized release of anti-inflammatory agents or biologics for chronic dermatoses such as psoriasis and atopic dermatitis, reducing dosing frequency and improving patient adherence. Hybrid physical–chemical enhancement: combining nanocarriers with physical permeation enhancers (microneedles, iontophoresis, low-frequency ultrasound, laser ablation) enables the transdermal delivery of macromolecules that otherwise cannot cross the stratum corneum, including peptides, proteins and nucleic acid therapeutics. Such hybrid approaches are being explored for systemic administration of biologics and for gene-editing interventions in skin disorders [134]. Also, organ- and cell-specific targeting ligand-decorated lipid nanomedicines, which have become standard in oncology and vaccine delivery, are now being adapted for dermatologic targets. By attaching antibodies or peptides that recognize melanoma-associated antigens or cutaneous T-cell lymphoma markers, nanocarriers can achieve selective uptake by malignant skin cells while sparing healthy tissue [135]. Integrative perspective: comprehensive studies of transdermal drug delivery systems stress that the convergence of nanotechnology, advanced biomaterials, micro-device engineering and personalized-medicine strategies will be essential to unlock the full therapeutic potential of skin-based delivery platforms.

## 11. Comparative Analysis of Transdermal Nanocarrier Systems

Many nanocarriers, such as liposomes, ethosomes, transfersomes, niosomes, polymeric nanoparticles, micelles, nanogels and SLNs, are used for transdermal drug delivery. Ethosomes and transfersomes are very good at drug loading, while polymeric nanoparticles and niosomes are more stable. However, transfersomes are more deformable, thus improving skin penetration, while ethosomes enhance skin retention. In addition, polymeric nanoparticles enable targeted delivery, and niosomes, SLNs and polymeric systems have better industrial scalability. Although advanced systems are superior for delivery, liposomes and niosomes are more established in the clinic [4]. For nanocarriers to be useful, they need to achieve an optimal balance between therapeutic performance and stability, scalability and clinical feasibility, as highlighted in Table 7.

## 12. Clinical Translation and Marketed Nanocarrier Products

Several nanotechnology-enabled products have successfully reached the market, demonstrating the translational potential of advanced drug delivery systems. Liposomal formulations remain among the most clinically established nanocarrier platforms because of their ability to improve drug stability, reduce toxicity and enhance tissue targeting. Examples include Doxil^®^, the first United States Food and Drug Administration-approved nanomedicine, and AmBisome^®^, a liposomal amphotericin B formulation that demonstrated the clinical feasibility of nanocarrier-mediated drug delivery. In dermatology and cosmeceuticals, lipid nanoparticles, nanoemulsions and liposomal formulations have been incorporated into commercial products designed to improve skin penetration and drug retention. Despite these successes, relatively few polymeric and hybrid nanocarrier systems have achieved regulatory approval because of challenges related to large-scale manufacturing, long-term stability, batch-to-batch reproducibility and regulatory classification. Continued collaboration between formulation scientists, clinicians and regulatory agencies will be essential to accelerate the clinical translation of next-generation dermal nanocarriers [138,139,140].

## 13. Conclusions

Nanotechnology-based drug delivery systems are transforming dermal and transdermal therapies by addressing shortcomings of conventional formulations. The stratum corneum acts as a barrier to drug permeation, thus limiting many therapeutically relevant agents, especially hydrophilic and high-molecular-weight compounds. Nanocarriers such as liposomes, ethosomes, transfersomes, nanoparticles, micelles and solid lipid hybrids enhance the solubility, stability and skin penetration of drugs through different permeation routes and allow localized and systemic delivery. These systems can offer improved therapeutic outcomes in preclinical dermatology models, reduced dose frequency and systemic toxicity and thus improved patient compliance. Advanced strategies like stimulus-responsive systems and microneedle incorporation have been developed to improve controlled drug release and transdermal flux of macromolecules. While nanocarrier-based systems offer several advantages over traditional formulations, their mechanisms of skin penetration are complex and often limited to enhancing local drug deposition rather than facilitating the direct passage of intact carriers into systemic circulation. Moreover, challenges such as large-scale manufacturing, formulation stability, regulatory framework and the need for high-quality clinical trials still remain. Moreover, the majority of products on the market today are still in the preclinical or early clinical stages, indicating a gap between experimental success and practical application. Therefore, it is recommended to develop smart, multi-functional and personalized systems by integrating wearable devices and artificial intelligence for real-time monitoring and drug release. In addition, researchers, clinicians and regulators need to work together to solve translational problems. Nanocarrier-based transdermal systems are necessary to improve dermatologic therapy by moving to safer and more effective treatments that bridge research and clinical practice. Future research should focus on advancing clinically relevant, bio-based and hybrid polymeric nanocarrier formulations by addressing translational barriers and regulatory requirements.

## Figures and Tables

**Table 1:** Advantages of nanocarriers in dermal and transdermal delivery.

Rationale/Benefit	Key Mechanistic Basis	Representative Nanocarriers	References
Enhanced solubility and loading of poorly soluble or unstable drugs	High surface area and hydrophobic cores (lipid bilayers, solid-lipid matrices, polymeric cores) solubilize lipophilic or labile actives and protect them from oxidation and enzymatic degradation, enabling higher loading than conventional creams or gels.	Solid lipid nanoparticles (SLNs), nanostructured lipid carriers (NLCs), liposomes, polymeric nanoparticles	[25,26,27,28]
Improved skin penetration and deposition	Vesicular deformability, lipid-fluidizing excipients (ethanol, surfactants) and follicular accumulation allow carriers to traverse or bypass the ordered stratum–corneum lipid matrix, enhancing entry into viable epidermis and dermis.	Transfersomes, ethosomes, transethosomes, niosomes, oleic acid vesicles, lipid–polymer hybrids, nanogels	[29,30,31,32,33]
Controlled and sustained release	Diffusion from polymer or lipid matrices and gradual carrier disassembly prolong drug release, flattening and extending, which reduces dosing frequency and concentration swings.	Poly lactic-co-glycolic acid (PLGA) and other polymerosomes nanoparticles, polymeric micelles, liposomes, SLNs/NLCs	[34,35,36]
Targeting specific skin compartments or cell types	Tuning particle size, charge and surface ligands directs carriers to particular routes (intercellular vs. follicular) and cell populations (keratinocytes, immune cells, tumor cells).	Ligand-decorated polymeric NPs, hyaluronic acid (HA)-based nanogels, lipid–polymer hybrids, immune-cell-targeted vesicles	[37,38,39,40]
Reduced systemic toxicity and optional systemic delivery	High local deposition in diseased skin limits systemic absorption; when desired, enhanced flux across the barrier enables lower total doses for systemic therapy.	Corticosteroid nanocarriers, ethosomes, transethosomes, transfersomes	[38,39,40,41]
In vivo evidence of superior efficacy	Improved retention, cell uptake and targeting translate into better clinical and histological outcomes versus free drugs at similar or lower doses.	Liposomal NSAIDs, polymeric micelles, SLNs/NLCs, polymeric nanoparticles (NPs)	[42,43,44,45]

**Table 2:** Major classes of nanocarriers for dermal and transdermal delivery.

Class	Representative Systems	Features Relevant to Skin Delivery	Typical Therapeutic Uses	References
Vesicular systems	Liposomes; ethosomes; transfersomes; transethosomes; niosomes; oleic acid vesicles	• Bilayer or surfactant-rich vesicles can encapsulate both hydrophilic and lipophilic drugs. • Deformable or ethanol-rich variants help fluidize stratum–corneum lipids, improving drug permeation. • Some vesicles serve dual roles as carriers and penetration enhancers.	Topical corticosteroids, calcineurin inhibitors, antivirals, antifungals, anti-acne agents, chemotherapeutics and hormones for both local and transdermal therapy.	[51]
Polymeric systems	Polymeric nanoparticles, (e.g., PLGA, chitosan); polymeric micelles; nanogels especially (chitosan or HA-based)	• Solid or self-assembled colloids possess adjustable size and surface charge. • Facilitate high loading capacity for poorly soluble active compounds. • Allow for controlled release and surface functionalization using targeting ligands. • Improve adhesion and can transiently open tight junctions.	Psoriasis (methotrexate, calcipotriol, siRNA), acne and rosacea (antibiotics, anti-androgens), skin cancers (5-FU, imiquimod, photosensitizers) and cosmetic/anti-aging actives.	[52]
Hybrid/advanced systems	Solid lipid nanoparticles (SLNs); nanostructured lipid carriers (NLCs); lipid–polymer hybrids; nanocarriers in dissolving or coated microneedles; smart stimuli-responsive systems.	• Combines advantages of lipid occlusion, hydration and biocompatibility. • Integrates polymers for mechanical stability and programmable release. • Integrated into microneedle arrays to bypass the stratum corneum. • Designed to respond to pH, enzymes or ROS in diseased skin.	Long-acting delivery for psoriasis, atopic dermatitis, infections, alopecia, vaccines and systemic small molecules or biologics via transdermal microneedle patches.	[53]

**Table 3:** Characteristics and recent applications of polymer-based nanocarriers for skin and transdermal drug delivery.

Polymer-Based Nanocarrier	Key Properties	Advantages	Representative Findings	References
Chitosan	Biodegradable, cationic, mucoadhesive	Opens tight junctions, enhances skin permeation, prolongs residence time	Tacrolimus-loaded chitosan nanoparticles improved skin deposition and reduced therapeutic dose in atopic dermatitis	[65]
Hyaluronic Acid (HA)	Biocompatible, highly hydrating, CD44-targeting	Enhances skin hydration, receptor-mediated uptake, improved retention	HA-curcumin micelles increased skin permeation and retention; HA nanoparticles delivered via microneedles improved psoriasis therapy	[25,66]
Alginate	Anionic, gel-forming, non-toxic	Controlled and pH-responsive drug release	Curcumin-loaded alginate nanoparticles encapsulation efficiency and pH-dependent release behavior	[67,68]
Cellulose Derivatives	Film-forming, mechanically stable, sustained release	Controlled drug release and follicular targeting	Ethyl cellulose nanosponges promoted wound closure by day; dexamethasone nanoparticles penetrated into hair follicles	[69,70]
Gelatin	Biocompatible, biodegradable, collagen-derived	Controlled degradation and wound healing support	pH-responsive gelatin nanoparticles enabled complete drug release under chronic wound conditions and enhanced antimicrobial activity	[71,72]

**Table 4:** Localized skin focused applications of nanocarrier systems in dermatologic disorders.

Dermatologic Indication	Nanocarrier Type(s)	Representative Therapeutic(s)	Primary Benefit Over Conventional Dosage Forms	Stage of Evidence	References
Psoriasis	Liposomes, solid-lipid nanoparticles (SLNs), polymeric nanoparticles, polymeric micelles, HA-nanoparticle-coated microneedles	Methotrexate, calcipotriol, tacrolimus, siRNA, estradiol	Higher drug accumulation in inflamed plaques, reduced PASI-like scores, suppression of IL-17A/IL-23, lower systemic exposure	Preclinical (in vitro, ex vivo, animal models)	[77]
Dermatitis/eczema	SLNs, nanostructured lipid carriers (NLCs), polymeric nanoparticles	Corticosteroids, calcineurin inhibitors	Prolonged epidermal residence, dose-sparing, decreased risk of steroid-induced atrophy	Preclinical (in vitro, animal models)	[101]
Acne/rosacea	Transfersomes, ethosomes, polymeric nanoparticles	Clindamycin, spironolactone	Targeted delivery to hair follicles, reduced systemic absorption, lower chance of antimicrobial resistance	Preclinical	[102]
Fungal infections	Liposomal vesicles, oleic acid vesicles	Antifungal agents (e.g., terbinafine)	Enhanced penetration into stratum corneum and nail bed, improved cure rates in resistant infections	Preclinical (in vitro, ex vivo skin/nail models)	[103]
Skin cancers and precancers	Liposomes, transfersomes, polymeric nanoparticles	5-Fluorouracil, imiquimod, photosensitizers	Elevated intra-tumoral concentrations, minimized systemic toxicity, better histologic outcomes	Preclinical (in vitro, animal models)	[104]
General anti-inflammatory topical therapy	Organic lipid-based or polymeric nanocarriers (excluding inorganic)	Various corticosteroids, NSAIDs	Superior skin compatibility, consistent efficacy in animal dermatitis models	Preclinical (animal dermatitis models)	[105]

**Table 5:** Nanocarrier-based transdermal strategies for systemic drug delivery.

Strategy	Nanocarrier Platform(s)	Example Payload(s)	Distinct Advantage for Systemic Delivery	References
Flux-boosting vesicles	Ethosomes, transfersomes, transethosomes	Small-molecule antihypertensives, analgesics	Disruption of SC lipid order higher permeation rates	[110]
Protection and transport of macromolecules	Liposomes, polymeric nanoparticles, lipid–polymer hybrids (often paired with microneedles, iontophoresis, electroporation or ultrasound)	Peptides, proteins, siRNA, vaccines	Shields cargo from enzymatic degradation; physical adjuncts create transient pathways for large entities	[111]
Sustained-release transdermal depots	SLNs, NLCs, polymeric micelles incorporated into patches or microneedle arrays	Hormones, chronic-pain agents, cardiovascular drugs	Enables days-to-weeks of steady plasma levels, eliminating frequent injections	[112]
Smart, on-demand platforms	Nanocarriers integrated with wearable sensors or stimuli-responsive matrices (pH, temperature, ROS)	Various systemic therapeutics	Real-time dosing adjustments based on physiological signals; supports precision medicine	[113]

**Table 6:** Ongoing clinical trials for various skin drug delivery systems for dermatologic therapy.

No.	Study Title	Indication	Phase	Intervention	Formulation Type (Route of Administration)	Outcome Measures	Status	Sponsor	Study Type	NCT Number
1	Global Healthcare Study on Atopic Dermatitis	Atopic dermatitis (AD) atopic dermatitis (eczema)	NA	Not provided	Topical	Eczema Area and Severity Index (EASI), clinician-assessed measure of atopic dermatitis severity based on extent and severity of eczema signs	Recruiting	Julia Tatjana Maul	Observationa	NCT07659756
2	Corticosteroids for Doxorubicin Liposome-induced hand-foot-skin reactions	Breast cancer	Phase 3	Dexamethasone (2 mg QD, d1-5) | Doxorubicin hydrochloride liposome injection	Oral tablets and injection (intravenous infusion)	Number of participants with hand-foot syndrome (HFS) as assessed by CTCAE v5.0	Recruiting	Fudan University	Interventional	NCT07362914
3	A Phase 2b Dose-Ranging Study to Evaluate the Efficacy and Safety of ENV-294 in Adults with Moderate to Severe Atopic Dermatitis	Atopic dermatitis	Phase 2	ENV-294, other (placebo)	Atopic	Percent change in eczema area and severity index (EASI) score, baseline to week 12	Not yet recruiting	Enveda therapeutics	Interventional	NCT07643766
4	Evaluation of a Non-Invasive Device for Early Detection of Atopic Dermatitis Flares	Anatomic Stage III/IV Breast cancer AJCC v8 Atopic dermatitis	NA	Nevisense electrical impedance spectroscopy	Device	To assess the relationship between EIS measurements of skin and AD severity through a variety of patient-reported outcome measures	Recruiting	Castle Biosciences Incorporated	Observational	NCT07645820
5	Pilot Study: Oral Treatment of American Tegumentary Leishmaniasis (Cutaneous and Mucosal Forms) in the Elderly	Leishmaniasis (Brazilian)| Leishmaniasis, Mucocutaneous	Phase 2/ Phase 3	Miltefosine 50 mg, Pentoxifylline 400 mg Liposomal Amphotericin B	Oral capsules, oral tablets and injection (intravenous infusion)	Cure, complete healing of all lesions	Recruiting	University of Brasilia	Interventional	NCT06040489
6	Role of Minoxidil in Alopecia Areata Transepidermal Drug Delivery of Minoxidil Via Either Fractional Carbon Dioxide Laser or Microneedling Versus Its Topical Nanoparticles Preparation for Treatment of Alopecia Areata	Alopecia Areata	NA	Fractional CO_2_ laser, Derma pen (microneedling) Niosome minoxidil	Topical nanosomal gel, device (laser), device (microneedling)	Regrowth scale	Unknown status	Assiut University	Interventional	NCT05587257
7	The Therapeutic Effect of Curcumin in Nanogels Compared to 0.1% FAO in the Management of Oral Lichen Planus	Oral lichen planus	NA	Curcumin in Nanogels, 0.1% Fluocinolone Acetonide Oral Paste	Topical gel, topical paste	Change in OLP-DAS Score, evaluation of change in Oral Lichen Planus-Disease Activity Scale	Recruiting	Chulalongkorn University	Interventional	NCT06932848
8	A Phase Ib/II Study of HL-300 Ointment in Patients with Mild to Moderate Atopic Dermatitis	Atopic dermatitis	Phase 1/Phase 2	HL-300	Topical	Eczema area and severity index (EASI)	Not Yet Recruiting	Hangzhou Highlightll Pharmaceutical Co., Ltd.	Interventional	NCT07629778
9	Dermocosmetic Evaluation of Propolis Ointments in Atopic-Prone Dry Skin	Dry skin, atopic dermatitis, xerosis dermatitis	NA	Ethanolic extract of propolis, crude propolis, vehicle ointment	Ointment	Pruritus Visual Analog Scale (VAS)	Active not recruiting	Manara University	Interventional	NCT07618234
10	A Prospective Study on the Clinical Value of Skin Test for Oxaliplatin Hypersensitivity Reaction and Its Correlation with Biomarkers	Hypersensitivity reaction	NA	Oxaliplatin, Saline (0.9% NaCl)	Topical	Sensitivity and specificity	Not yet recruiting	Fujian Cancer Hospital	Interventional	NCT07613905
11	A First-in-human Study to Investigate Single Doses of DCY636 in Healthy Volunteers and Multiple Doses in Participants with Moderate to Severe Atopic Dermatitis	Dermatitis, atopic	Phase 1	DCY636	Topical	Incidence of adverse events (AEs) and serious adverse events (SAEs)	Recruiting	Novartis Pharmaceuticals	Interventional	NCT07604324
12	Phase 1 Study of VELGRAFT, a Living Cellular Construct, in the Management of Chronic Diabetic Foot Ulcers Which Have Attained Granulation Tissue	Diabetic Foot Ulcer (DFU), Granulation of Chronic Diabetic Wounds	Phase 1	VELGRAFT (allogenic cell-based product containing MSCs on chitosan-gelatin matrix)	Topical application (biological cellular dressing/matrix)	Incidence of Treatment-Emergent Adverse Events (Safety and Tolerability)	Not Yet Recruiting	Ayu, Inc.	Interventional	NCT07498218
13	Evaluating the Efficacy of FoundationDRS Solo in Addition to Standard of Care for the Treatment of Non-healing Diabetic Foot Ulcers	Diabetic Foot Ulcer (DFU)	NA	FoundationDRS Solo, Standard of Care	Topical application (biodegradable chitosan-chondroitin sulfate wound scaffold/dressing)	Incidence of Complete Wound Closure	Not Yet Recruiting	Samaritan Biologics	Interventional	NCT07290673
14	PMCF Investigation of Medical Device ChitoCare Medical	Dermatitis, acne, Conditions After Scars	NA	ChitoCare medical Wound Healing Gel, ChitoCare medical Healing Spray	Topical gel, topical spray	Significant improvement in Vancouver Scar Scale (VSS)	Recruiting	Primex ehf	Observational	NCT06850389
15	Photodynamic Therapy and Topical Antifungal for Onychomycosis in Patients with Diabetes	Dermatologic Disease, Onychomycosis of Toenail, Diabetic Foot	NA	Photodynamic therapy	Combination product (topical photosensitizer + light-activation device)	Assess the incidence of treatment-related adverse events	Active Not Recruiting	Universidad Complutense de Madrid	Interventional	NCT06485050
16	A Phase 2b Study of the Effects of Camoteskimab in Adults with Moderate-to-Severe Atopic Dermatitis	Eczema Atopic Dermatitis	Phase 2	Camoteskimab	Topical dematitis	Percentage change from baseline in Eczema Area and Severity Index (EASI)	Recruiting	Apollo Therapeutics Ltd.	Interventional	119 NCT07599813
17	Using a Contact Dermatitis Model with Biologic Medications to Study Skin Inflammation	Skin inflammation, Allergic Contact Dermatitis	Phase 2	Squaric Acid Dibutyl Ester	Topical solution (contact sensitizer)	Single-cell multiomics data collection (RNAseq, CITEseq, TCRseq)	Recruiting	Wei-Che Ko	Interventional	NCT05535738

NA: Not applicable.

**Table 7:** Comparative analysis of transdermal nanocarrier systems.

System	Drug Loading	Stability	Deformability	Targeting	Skin Retention	Transdermal Flux	Irritation Risk	Scalability	Clinical Maturity	References
Liposomes	Moderate	Low–Moderate	Low	Possible	Moderate	Moderate	Low–Med	Moderate	Moderate	[4]
Niosomes	Moderate	High	Moderate	Possible	Moderate	Moderate	Low	High	Moderate	
Ethosomes	High	Moderate	High	Limited	High	High	Moderate	Moderate	Low	[22]
SLNs	Moderate	High	Low	Possible	Moderate	Moderate	Low	High	Low–Moderate	
Transfersomes	High	Moderate	Very High	Limited	High	Very High	Moderate	Moderate	Low	[136]
Polymeric NPs	High	High	Low	High	High	Moderate	Low	High	Low	
Micelles	Low–Moderate	Moderate	Low	Possible	Low	Low	Low	High	Low	[76]
Nanogels	High	Moderate	Moderate	Possible	High	Moderate	Low	Low	Low	[137]

## Data Availability

No new data were created or analyzed in this study. Data sharing is not applicable to this article.
